# Deep Learning Neural Networks to Predict Serious Complications After Bariatric Surgery: Analysis of Scandinavian Obesity Surgery Registry Data

**DOI:** 10.2196/15992

**Published:** 2020-05-08

**Authors:** Yang Cao, Scott Montgomery, Johan Ottosson, Erik Näslund, Erik Stenberg

**Affiliations:** 1 Clinical Epidemiology and Biostatistics School of Medical Sciences Örebro University Örebro Sweden; 2 Clinical Epidemiology Division Department of Medicine Karolinska Institutet Stockholm Sweden; 3 Department of Epidemiology and Public Health University College London London United Kingdom; 4 Department of Surgery Faculty of Medicine and Health Örebro University Örebro Sweden; 5 Division of Surgery, Department of Clinical Sciences Danderyd Hospital Karolinska Institutet Stockholm Sweden

**Keywords:** projections and predictions, deep learning, computational neural networks, bariatric surgery, postoperative complications

## Abstract

**Background:**

Obesity is one of today’s most visible public health problems worldwide. Although modern bariatric surgery is ostensibly considered safe, serious complications and mortality still occur in some patients.

**Objective:**

This study aimed to explore whether serious postoperative complications of bariatric surgery recorded in a national quality registry can be predicted preoperatively using deep learning methods.

**Methods:**

Patients who were registered in the Scandinavian Obesity Surgery Registry (SOReg) between 2010 and 2015 were included in this study. The patients who underwent a bariatric procedure between 2010 and 2014 were used as training data, and those who underwent a bariatric procedure in 2015 were used as test data. Postoperative complications were graded according to the Clavien-Dindo classification, and complications requiring intervention under general anesthesia or resulting in organ failure or death were considered serious. Three supervised deep learning neural networks were applied and compared in our study: multilayer perceptron (MLP), convolutional neural network (CNN), and recurrent neural network (RNN). The synthetic minority oversampling technique (SMOTE) was used to artificially augment the patients with serious complications. The performances of the neural networks were evaluated using accuracy, sensitivity, specificity, Matthews correlation coefficient, and area under the receiver operating characteristic curve.

**Results:**

In total, 37,811 and 6250 patients were used as the training data and test data, with incidence rates of serious complication of 3.2% (1220/37,811) and 3.0% (188/6250), respectively. When trained using the SMOTE data, the MLP appeared to have a desirable performance, with an area under curve (AUC) of 0.84 (95% CI 0.83-0.85). However, its performance was low for the test data, with an AUC of 0.54 (95% CI 0.53-0.55). The performance of CNN was similar to that of MLP. It generated AUCs of 0.79 (95% CI 0.78-0.80) and 0.57 (95% CI 0.59-0.61) for the SMOTE data and test data, respectively. Compared with the MLP and CNN, the RNN showed worse performance, with AUCs of 0.65 (95% CI 0.64-0.66) and 0.55 (95% CI 0.53-0.57) for the SMOTE data and test data, respectively.

**Conclusions:**

MLP and CNN showed improved, but limited, ability for predicting the postoperative serious complications after bariatric surgery in the Scandinavian Obesity Surgery Registry data. However, the overfitting issue is still apparent and needs to be overcome by incorporating intra- and perioperative information.

## Introduction

### Background

Obesity is one of today’s most important public health problems worldwide. With no changes in the current trends, the estimated prevalence of severe obesity (BMI greater than 35 kg/m^2^) will reach 9% for women and 6% for men within a few years [1]. Obesity is associated with an increased risk of several conditions and diseases, such as type 2 diabetes, heart disease, and many more, and imposes a major growing threat for global public health [2]. It is a serious chronic condition that should be prevented and treated as early as possible [3]. Although medical weight management and pharmacotherapy are effective options, modern bariatric surgery offers one of the best chances for long-term weight loss and the resolution of comorbidity risk [4].

Although modern bariatric surgery is considered to be ostensibly safe, serious complications and mortality still occur in some patients [5-7]. Thus, preoperative risk assessment is one of the most important components of surgical decision making. Numerous studies have attempted to predict the risk for complications after bariatric surgery. Some studies developed new models based on national databases [5-9], and other studies applied the obesity surgery mortality risk score, although its accuracy for prediction is still unclear [7,10-14]. In recent years, the potential of addressing public health challenges and advancing medical research through the increasing amount of information regarding symptoms, diseases, and treatments, in parallel with the challenges inherent in working with such sources, are being recognized [15]. A variety of machine learning (ML) methods, including artificial neural networks [16], decision trees [17], Bayesian networks [18], and support vector machines [19], have been widely applied with the aim of detecting key features of the patient conditions and modeling the disease progression after treatment from complex health information and medical datasets. The application of different ML methods in feature selection and classification in multidimensional heterogeneous data can provide promising tools for inference in medical practices [20,21]. These highly nonlinear approaches have been utilized in medical research for the development of predictive models, resulting in effective and accurate decision making [22-24].

In our previous studies, conventional statistical models [8] and ML methods [9] were used to predict the likelihood of serious complication after bariatric surgery. Although some potential risk factors, such as revision surgery, age, lower BMI, larger waist circumference (WC), and dyspepsia, were associated with a higher risk for serious postoperative complications by the multivariate logistic regression model, the sensitivity of the model for prediction was quite low (<0.01) [8]. When comparing 29 ML algorithms, we found that overfitting was still the overwhelming problem even though some algorithms showed both high accuracy >0.95 and an acceptable area under curve (AUC) >0.90 for the training data [9]. Despite these unfavorable aspects, our study suggests that deep learning neural networks (DLNNs) have the potential to improve the predictive capability and deserve further investigation.

Although there is increasing evidence that the use of ML methods can improve our understanding of postoperative progression of bariatric surgery [25-30], few studies have used DLNNs to predict the prognosis after bariatric surgery, and validation is needed to select a proper method in clinical practice.

### Objectives

The aim of this study was to examine whether serious postoperative complications of bariatric surgery can be predicted preoperatively using DLNNs based on the information available from a national quality registry. We used the data from the Scandinavian Obesity Surgery Registry (SOReg) to examine the performance of 3 widely used DLNNs.

## Methods

### Patients and Features

The SOReg covers virtually all bariatric surgical procedures performed in Sweden since 2010 [31]. Patients who were registered in the SOReg between 2010 and 2015 were included in this study. Information for the patients who underwent a bariatric procedure between 2010 and 2014 was used as training data, and information from those in 2015 was used as test data. Postoperative complications were graded according to the Clavien-Dindo classification, and complications requiring intervention under general anesthesia or resulting in organ failure or death were considered serious (ie, grade 3b or higher) [32]. The primary outcome was serious complications occurring within the first 30 days after bariatric surgery. Details of the data have been described elsewhere [8,9]. Briefly, 37,811 and 6250 patients were used as the training data and test data, with incidence rates of serious complication of 3.2% (1220/37,811) and 3.0% (188/6250), respectively. In general, the patients with and without serious complication were balanced in baseline demographic characteristics and comorbidity in the 2 datasets, except that the patients with serious complications were a little older (mean 42.9 vs 41.2 years; *P*<.001) and had greater WCs (mean 126.2 vs 123.2 cm; *P*=.009) compared with those without serious complications in the test dataset [9]. Except for the outcome variable, 16 features of the patients were used for ML, including 5 continuous features (age, hemoglobin A_1c_ [HbA_1c_], BMI, WC, and operation year) and 11 dichotomous features (sex; sleep apnea; hypertension; diabetes; dyslipidemia; dyspepsia; depression; musculoskeletal pain; previous venous thromboembolism; revisional surgery; and the outcome, serious postoperative complications).

The Regional Ethics Committee in Stockholm approved the study (approval number: 2013/535-31/5).

### Deep Learning Neural Networks

Three supervised DLNNs were applied and compared in our study, comprising multilayer perceptron (MLP), convolutional neural network (CNN), and recurrent neural network (RNN) models. For the MLP model, we used 4 dense layers and 2 dropout layers. The initial computation units for the dense layers were set to 15, 64, 64, and 128, and dropout rate was set to 0.5 for the 2 dropout layers (Multimedia Appendix 1). The rectified linear unit (relu) activation function was used for the 3 dense layers, and the sigmoid activation function was used for the last dense layer. The binary cross-entropy loss function and the root mean square propagation optimizer were used when compiling the model [33].

In the initial CNN, we used a 7-layer model with 2 one-dimensional (1D) convolution layers (with 10 filters for each), 2 1D max pooling layers, 1 flatten layer, and 2 dense layers (with 1000 computation units). The relu activation function was used for the 2 1D convolution layers and the first dense layers, and the sigmoid activation function was used for the last dense layer. The binary cross-entropy loss function and the adaptive moment estimation (Adam) optimizer were used when compiling the model (Multimedia Appendix 2) [34].

In view of the temporal feature of the data, we also used the RNN for prediction. To minimize computation time, the initial model only included 1 long short-term memory (LSTM) layer and 1 dense layer. The initial dimensionality of the LSTM layer was set to 32. To tackle overfitting, we randomly dropped out inputs and recurrent connections in the LSTM layer to break happenstance correlations in the training data that the layer was exposed to. The dropout rates for inputs and recurrent connections were set to 0.2. The activation functions for input connection and recurrent connection were hyperbolic tangent and hard sigmoid, respectively. The activation function for the dense layer was sigmoid. The binary cross-entropy loss function and the Adam optimizer were used when compiling the model.

### Feature Scaling

For the training data, the binary features were converted into dummy variables, and the continuous features were standardized to have mean 0 and SD 1 before they enter the model. For the test data, the continuous features were standardized using the corresponding means and standardizations from the training data. HbA_1c_ was log transformed before standardization because of its asymmetrical distribution. In sensitivity analysis, the normalizer and min-max scaler were also used to evaluate the influence of scalers on the models’ performance.

### Data Augmentation

As the incidence rate of serious complications is very low (only 3.2%), the extreme imbalance would result in serious bias in the performance metrics [35]. Therefore, we used the synthetic minority oversampling technique (SMOTE) to artificially augment the proportion of patients with serious complications. SMOTE generates a synthetic instance by interpolating the *m* instances (for a given integer value *m*) of the minority class that lies close enough to each other to achieve the desired ratio between the majority and minority classes [36]. In our study, a SMOTE dataset with a 1:1 ratio between the patients with and without serious complications was generated and used for training.

### Performance Metrics

The performances of the three neural networks were evaluated using accuracy, sensitivity, specificity, Matthews correlation coefficient (MCC) [37], and area under the receiver operating characteristic (ROC) curve. Terminology and derivations of the metrics are given in detail elsewhere [9]. A successful prediction model was defined as with an AUC greater than 0.7 [38,39].

### Validation During Model Training

To find optimal high-level parameters (such as the number, size, and type of layers in the networks) and lower-level parameters (such as the number of epochs, choice of loss function and activation function, and optimization procedure) in the DLNN models, the K-fold cross-validation method was used during the training phase. K-fold cross-validation is currently considered as a minimum requirement to handle the problems such as overfitting when applying only 1 single dataset in ML [40]. In this study, we split the training data into 5 partitions, instantiated 5 identical models, and trained each one on 4 partitions while evaluating the remaining partition. We then computed the average performance metrics over the 5 folds. In the end, the choice of the parameters was a compromise between the neural network’s performance and computation time: the model with a larger ratio of AUC to logarithmic computation time or no significant difference (*Δ*AUC≤0.01) found between the models’ performance. An example of parameters selection by grid searching for MLP model is given in Multimedia Appendix 3.

### Software and Hardware

The descriptive and inferential statistical analyses were performed using Stata 15.1 (StataCorp LLC, College Station). The DLNN models were achieved using packages scikit-learn 0.19.1 and Keras 2.1.6 in Python 3.6 (Python Software Foundation). The 95% CI of AUC was calculated using the package pROC in R 3.61 (R Foundation for Statistical Computing).

All the computation was conducted using a computer with the 64-bit Windows 7 Enterprise operating system (Service Pack 1), Intel Core TM i5-4210U CPU of 2.40 GHz, and 16.0 GB installed random access memory.

## Results

### Overview of the Performance of the 3 Deep Learning Neutral Networks

The incidence of serious complications after bariatric surgery in our study was 3.2%, which is similar to other studies [12,41]. The 3 DLNNs showed quite similar performance for our original training data, with specificity=1.00, sensitivity=0, and AUC≤0.6 (Table 1). Although the models’ specificity dropped when trained using SMOTE data, the sensitivity increased significantly from 0 to 0.97 in the MLP model and 0.70 in the CNN model (Table 1), and AUC also achieved an acceptable level (>0.7). The finding confirms our previous assumption that DLNNs trained by SMOTE data might have better performance in predicting serious complications after bariatric surgery [9]. However, the performance of the 3 DLNNs in the test data was still low; the highest AUC was only 0.23 for the MLP trained by the SMOTE data (Table 1). MCC measures indicate that the MLP trained by the SMOTE data showed promising prediction (MCC=0.44) for the training data; however, the performance of the 3 DLNNs was only slightly better than random prediction (MCC=0.02, 0.03, and 0.05 for MLP, CNN, and RNN, respectively) for the test data (Table 1).

**Table 1:** Performance metrics of the models.

Model	Training data					Test data				
	Accuracy	Specificity	Sensitivity	MCC^a^	AUC^b^ (95% CI)	Accuracy	Specificity	Sensitivity	MCC	AUC (95% CI)
MLP^c^	0.97	1.00	0.00	0.00	0.60 (0.59-0.61)	0.97	1.00	0.00	0.00	0.57 (0.55-0.59)
MLP^d^	0.68	0.39	0.97	0.44	0.84 (0.83-0.85)	0.84	0.82	0.23	0.02	0.54 (0.53-0.55)
CNN^e^	0.97	1.00	0.00	0.00	0.58 (0.56-0.60)	0.97	1.00	0.00	0.00	0.55 (0.54-0.56)
CNN^d^	0.63	0.56	0.70	0.26	0.79 (0.78-0.80)	0.95	0.97	0.06	0.03	0.57 (0.59-0.61)
RNN^f^	0.97	1.00	0.00	0.00	0.58 (0.57-0.59)	0.97	1.00	0.00	0.00	0.56 (0.55-0.57)
RNN^d^	0.58	0.66	0.49	0.15	0.65 (0.64-0.66)	0.91	0.93	0.14	0.05	0.55 (0.53-0.57)

^a^MCC: Matthews correlation coefficient.

^b^AUC: area under curve.

^c^MLP: multilayer perceptron.

^d^Trained using synthetic minority oversampling technique data.

^e^CNN: convolutional neural network.

^f^RNN: recurrent neural network.

### Performance of Multilayer Perception

**Figure 1:**
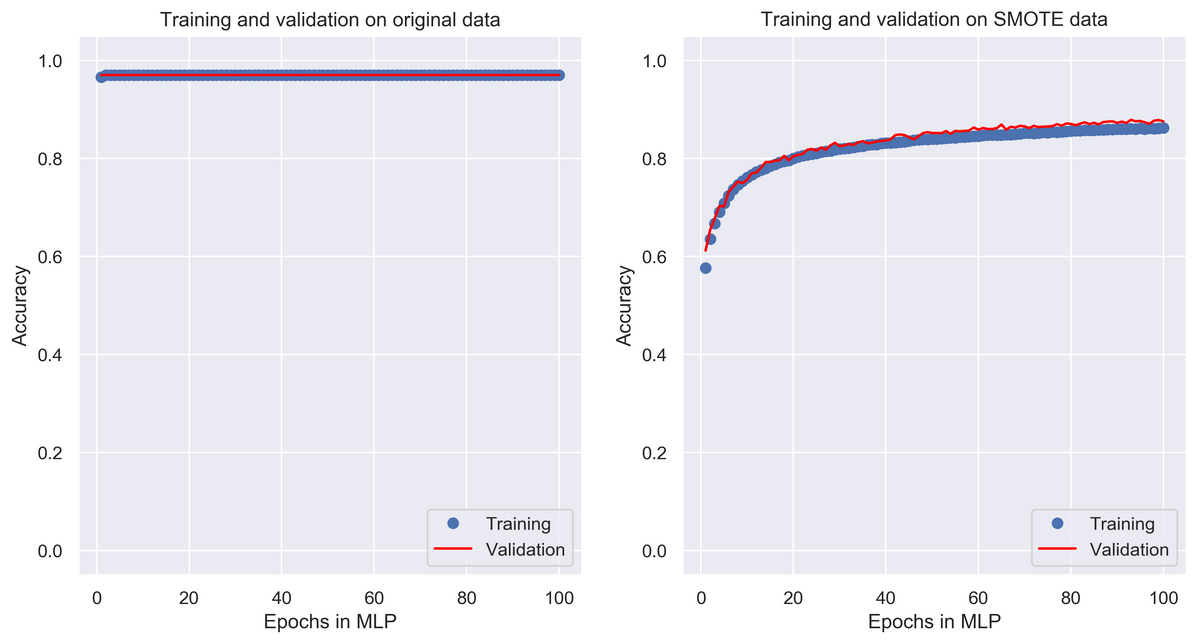
Change of accuracy with the number of epochs in multilayer perceptron. MLP: multilayer perceptron; SMOTE: synthetic minority oversampling technique.

There were myriad combinations of high- and low-level parameters used during model training, and most of them resulted in constant performance after given values. Therefore, we only show the trend of the MLP model’s accuracy with number of epochs for model training while keeping other parameters unchanged in Figure 1. When learning from the original data, the accuracy almost did not change along with the number of epochs, which was a constant value 0.968 (Figure 1, left panel). The reason is that the incidence rate of serious complications was only 3.2%; therefore, although the model always predicted a patient as having a serious complication, it achieved high accuracy (>0.96), whereas in the SMOTE data where the numbers of patients with and without serious complications are equal, the choice of number of epochs shows a significant influence on accuracy. When the epochs are less than 20, the accuracy is smaller than 0.8, and it approximates to 0.85 when epochs are greater than 80 and remains almost constant afterward (Figure 1, right panel). As the computing time is proportional to the number of epochs, we selected epochs 80 for model training.

**Figure 2:**
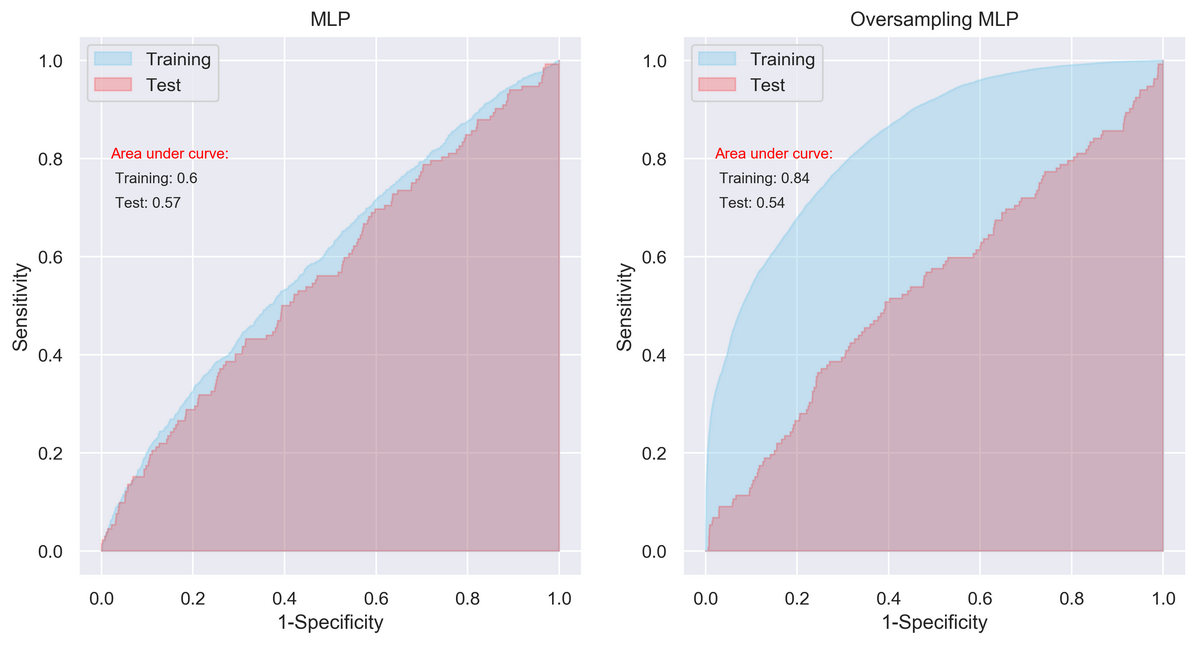
Area under curve of multilayer perceptron with initial setting. MLP: multilayer perceptron.

The performance of the MLP was not optimal for the original training data and test data. The AUCs were barely higher than a random guess, that is, 0.5, which were 0.60 (95% CI 0.59-0.61) and 0.57 (95% CI 0.55-0.59) for the training data and test data, respectively (Figure 2, left panel). When trained using the SMOTE data, the performance of the MLP improved notably, with an AUC of 0.84 (95% CI 0.83-0.85). However, its performance was still low for the test data, with an AUC of 0.54 (95% CI 0.53-0.55; Figure 2, left panel).

**Figure 3:**
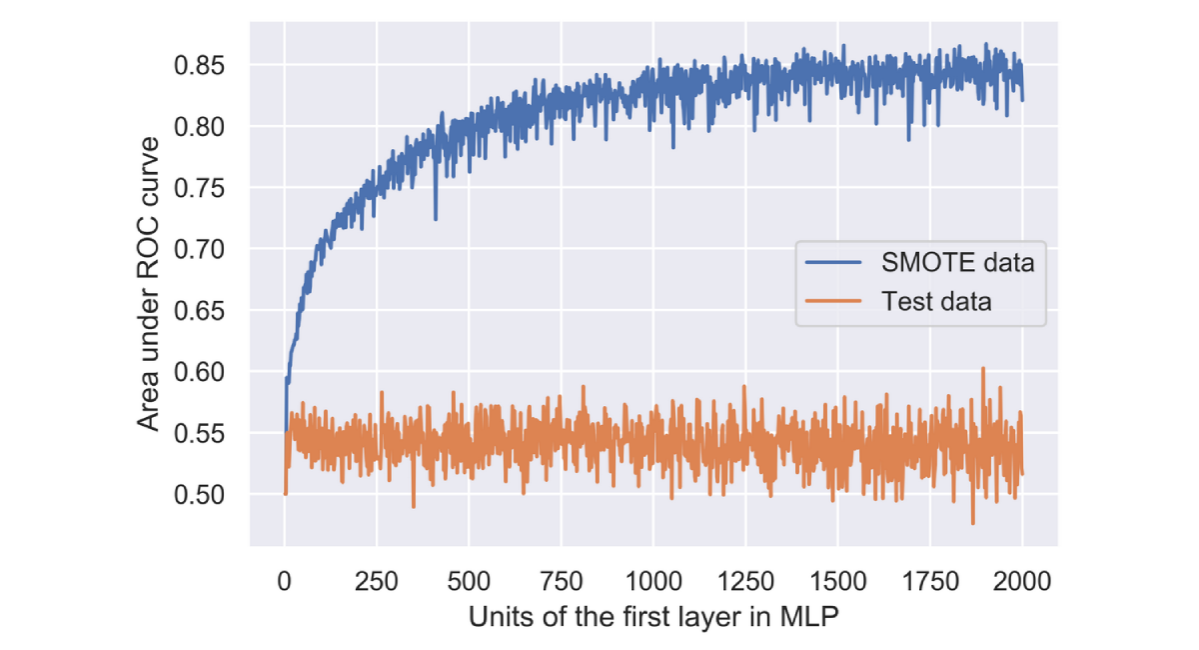
Performance of multilayer perceptron using the synthetic minority oversampling technique and test data with different numbers of computation units in the first hidden layer. MLP: multilayer perceptron; ROC: receiver operating characteristic; SMOTE: synthetic minority oversampling technique.

The performance of MLP was significantly influenced by the number of computation units in the SMOTE data but not in the test data. For example, when the computation units of the first layer ranged from 4 to 500, the AUC increased rapidly from 0.55 to 0.80. Within the range from 500 to 1000, the AUC increased slowly from 0.80 to 0.85 and kept fluctuating around 0.85 afterward (Figure 3). However, the AUC kept fluctuating around 0.55 in the test data no matter how many units were used (Figure 3).

### Performance of Convolutional Neutral Network

**Figure 4:**
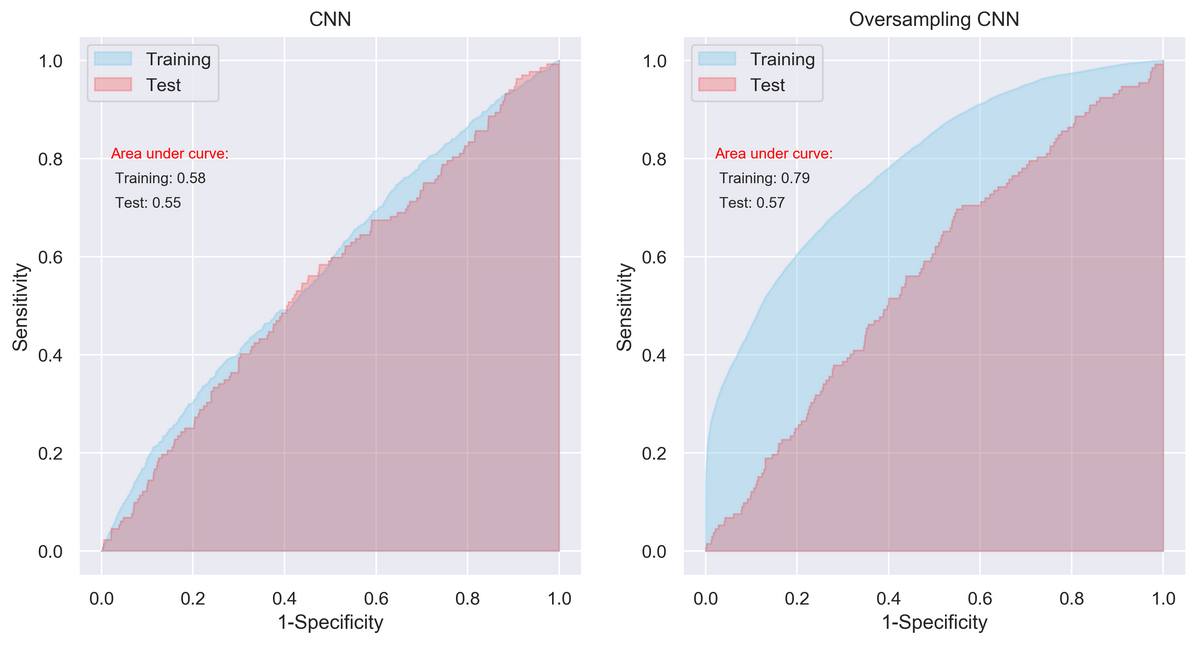
Area under curve of convolutional neural network with initial setting. CNN: convolutional neural network.

The performance of CNN appeared to be similar to that of MLP. The AUCs were 0.58 (95% CI 0.56-0.60) and 0.55 (95% CI 0.54-0.56) for the training data and test data, respectively (Figure 4, left panel). When trained using the SMOTE data, the AUCs were 0.79 (95% CI 0.78-0.80) and 0.57 (95% CI 0.59-0.61), respectively (Figure 4, right panel). Again, although the model’s performance seems to be improved significantly after training by the artificially balanced SMOTE data, its performance on the unseen test data still appears low.

**Figure 5:**
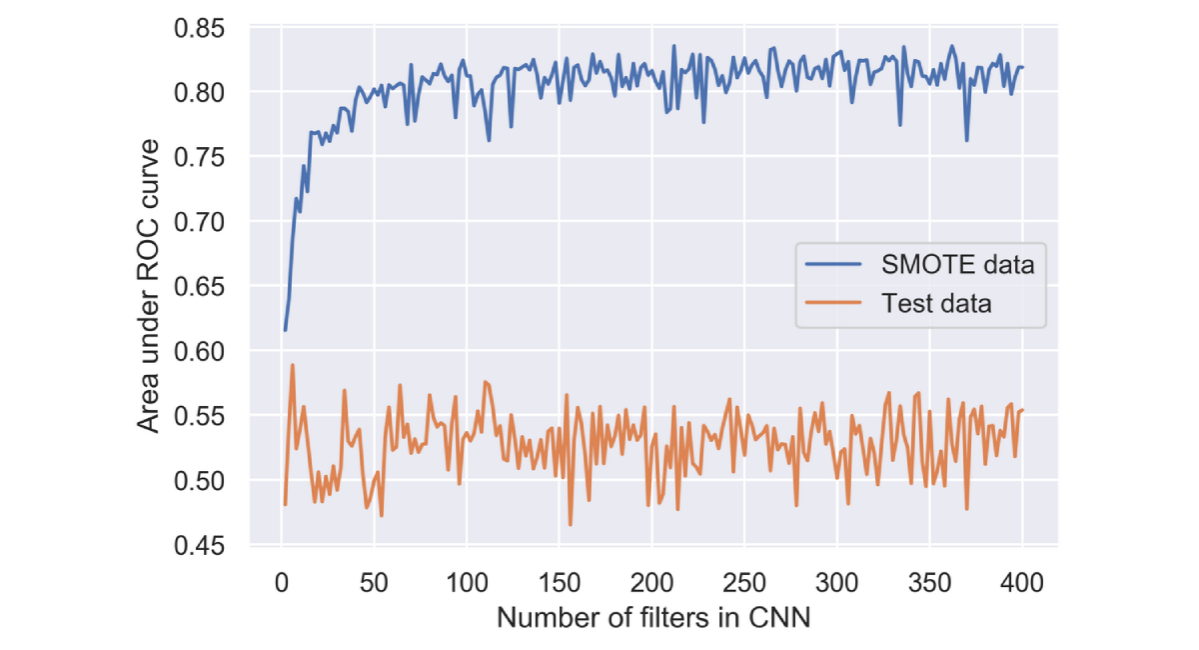
Performance of convolutional neural network using the synthetic minority oversampling technique and test data with different numbers of filters. CNN: convolutional neural network; ROC: receiver operating characteristic; SMOTE: synthetic minority oversampling technique.

The number of output filters in the convolution (or the dimensionality of the output space) has a significant influence on the CNN model’s performance in the SMOTE data but not in the training data and test data. The AUC of CNN increased rapidly from 0.63 to 0.80 when we set the number of filters from 5 to 50. However, the larger number of filters contributes no further improvement (Figure 5). The CNN model trained by the SMOTE data always gave an AUC around 0.52 in the test data (Figure 5).

### Performance of Recurrent Neutral Network

**Figure 6:**
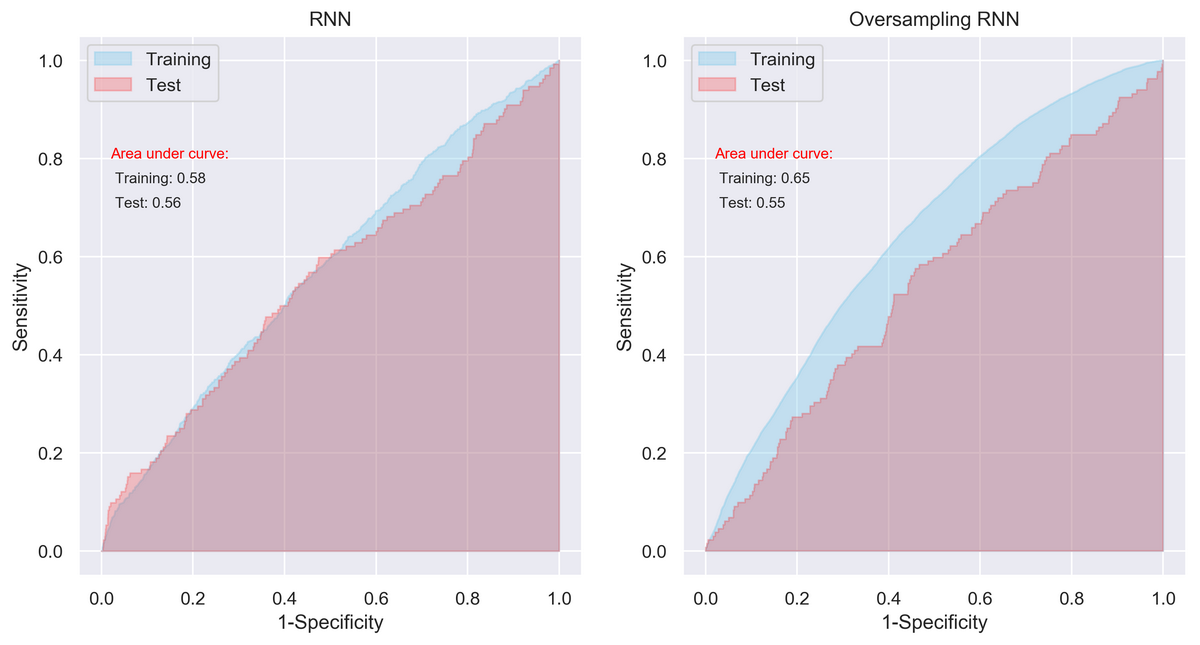
Area under curve of recurrent neural network with initial setting. RNN: recurrent neural network.

Compared with the MLP and CNN, the RNN showed even worse performance. AUCs of RNN for the original training data and test data were 0.58 (95% CI 0.57-0.59) and 0.56 (95% CI 0.55-0.57), respectively (Figure 6, left panel). For the SMOTE data, the AUC was only 0.65 (95% CI 0.64-0.66; Figure 6, right panel), which was significantly lower than those derived from MLP (AUC=0.83) and CNN (AUC=0.81). The AUC of RNN trained by the SMOTE data was only 0.55 (95% CI 0.53-0.57) for the test data.

**Figure 7:**
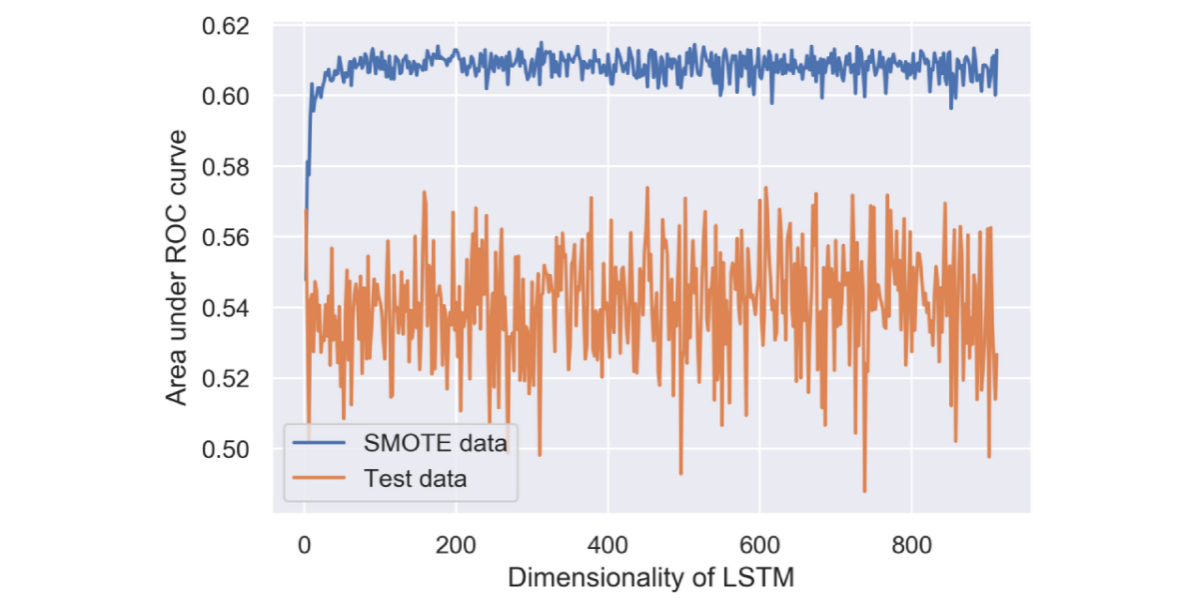
Performance of recurrent neural network using the synthetic minority oversampling technique and test data with different dimensionalities of long short-term memory. LSTM: long short-term memory; ROC: receiver operating characteristic; SMOTE: synthetic minority oversampling technique.

The performance of the RNN model was influenced by the dimensionality of the LSTM layer. The AUC changed from 0.50 to 0.60 rapidly when the dimensionality grew from 2 to 20 and kept fluctuating around 0.61 afterward (Figure 7). Although other hyperparameters, such as kernel initializer and regularizer, also had an influence on the RNN’s performance, their impacts were not as notable as the dimensionality of layer.

### Sensitivity Analysis and Computing Time

In the sensitivity analysis, we tried different scalers and optimizers in data preparation and model compiling, and we tried thousands of combinations of hyperparameters for each model using the exhaustive grid search method [42]. Although they showed more or less influence on the models’ performance, the influence was negligible compared with the exponentially increased computing time. Therefore, we only show the results of the model with the optimal hyperparameters in the figures above.

The computing time for the models was largely dependent on the number of DLNN layers and hyperparameter settings of the layers, number of epochs and batch size for training, and obviously software and hardware used. In our study, with the model structures and hyperparameters described above, the running time ranged from 82 seconds for the MLP model (computational units=64, epochs=80, batch size=128, and trained by original data) to more than 10 hours for the CNN model (filters=400, epochs=100, batch size=128, and trained by SMOTE data with cross-validation and grid search) on our computer.

## Discussion

### Principal Findings

Several studies have explored using ML methods to predict the risks after bariatric surgery. Razzaghi et al [27] evaluated 6 of the most popular classification methods to predict 4 common outcomes (diabetes, angina, heart failure, and stroke) using 11,636 patients from the Premier Healthcare Database of the United States. The study also applied the SMOTE technique to handle the imbalance issue in the data, and the results indicate that random forest and bagging methods outperform other methods [27]. However, the study did not test methods using outer unseen data. Therefore, the real performance of the methods is questionable. Thomas et al [28] predicted the long-term weight status after bariatric surgery in 478 patients using 8 neural networks. Their neural networks yielded an AUC of 0.77 to 0.78 in predicting weight loss success. However, the types of the neural networks used were not reported. It seems as if the authors only used 1 neural network but with different variables as input. Pedersen et al [25] used neural networks integrating clinical and genomic biomarkers for 268 patients to rank factors involved in type 2 diabetes remission after bariatric surgery, and Hayes et al [26] used the decision tree and the Naive Bayes to establish independent predictors for the resolution of type 2 diabetes in 130 patients. However, the sample sizes of both studies seem too small for nonlinear ML algorithms; therefore, models might only have a high internal validity but not external validity [43]. In our previous study, we trained and compared 29 basic ML algorithms using information from 37,811 patients to predict serious complications after bariatric surgery. Although several ensemble algorithms, such as random forest, gradient regression tree, and bagging k-nearest neighbor, showed favorable performance, the overfitting problem was apparent [9].

In this study, we applied and compared 3 DLNN models for predicting serious complications after bariatric surgery. MLP is the classical type of neural network, which consists of multiple layers of computational units. The layers are interconnected in a feedforward way, where the information moves only forward, that is, from input nodes, through hidden nodes and to output nodes, and the connections between the nodes do not form a cycle [44]. CNN is a regularized version of MLP, which was inspired by biological processes where the connectivity pattern between neurons resembles the organization of the animal’s visual cortex [45]. Although not specifically developed for nonimage data, CNN may achieve state-of-the-art results for classification prediction problems using time series data or sequence input data. The CNN input is traditionally two-dimensional but can also be changed to be 1D, allowing it to develop an internal representation of a 1D sequence. RNN is designed to work with sequence prediction problems and traditionally difficult to train, where connections between nodes form a directed graph along a temporal sequence, which allows it to exhibit temporal dynamic behavior. Unlike feedforward neural networks, RNN can use its internal state (memory) to process sequences of inputs. In effect, an RNN is a type of neural network that has an internal loop. It loops over time steps, and at each time step, it considers its current state at *t* and input at *t* and combines them to obtain the output at *t* [46]. RNN is traditionally difficult to train, but the LSTM network overcomes the problems of training a recurrent network and, in turn, has been perhaps the most successful and widely applied. Therefore, we adopted the LSTM network in this study. Regarding the choice of the number of layers in DLNNs, there is no universally agreed upon threshold, but most researchers in the field agree that DLNN has multiple nonlinear layers with a credit assignment path (CAP) >2, and Schmidhuber [44] considers CAP >10 to be very deep learning. To address a specific real-world predictive modeling problem, in general, we cannot analytically calculate the number of layers or the number of nodes in a DLNN and have to use systematic experimentation to discover what works best for our specific dataset.

Although the results from the MLP and CNN models seem promising in the SMOTE training data, the overfitting problem still exists, which was reflected in the poor performance of the 3 models in the test data (see Table 1 and the left panels in Figures 2, 4, and 6). It means that although we have identified potential risk factors related to serious complication after bariatric surgery at the population level [8], using current data available to predict whether an individual patient has a serious complication after bariatric surgery is still far from clinically applicable. Thus, despite using the most promising methods of ML, these results support a previous review of standard statistical methods for the prediction of complications in bariatric surgery, where models based only on factors known before surgery were insufficient to predict postoperative complications [47]. The main reason for this insufficiency is likely to be that all such methods are missing information on intraoperative adverse events, surgical experience, and perioperative optimization of patients, which are well-known important risk factors for adverse postoperative outcome [7,47-49].

We also noticed that the RNN performed worse than MLP and RNN for our data. The possible reason might be that the sequential pattern or temporal trend in our data cannot be represented by the features currently available in our data, or there is no dependency between the patients or events in the time-series. Even if the trend can be captured by the RNN, it might be weak, and the past status contributed noise rather than information to current status.

Although increasing the number of computational units in the layers or adding more layers may increase the model’s capacity, the trade-off between computational expensiveness and representational power is seen everywhere in ML. Limited by the computing power, we tried to avoid complicated networks such as applying multiple RNN layers or combining CNN and RNN, but it deserves investigation in the future with data having more variables and apparent temporal trend.

### Advantages and Limitations

Compared with previous studies, there are several advantages in our study. First, we used DLNNs rather than traditional ML techniques. The biggest advantage of DLNNs is that they try to learn high-level features from data in an incremental manner. They need less human domain expertise for hard-core feature extraction [50]. In contrast, in traditional ML techniques, most of the applied features have to be identified by domain experts to reduce the complexity of the data and make patterns more visible to learning algorithms to work [44]. Second, the study is based on a national quality register with extensive coverage (97%) of the target population, with a very high follow-up rate for the studied outcome. Therefore, on the one hand, the selection bias is minimized in the study, and the much larger sample size may ensure the external validity of the nonlinear ML algorithms. Third, we conducted different types of sensitivity analyses for feature scaling, hyperparameters optimization, and model compiling during data training, which ensure the efficiency and internal validity of our models. However, we also have to admit that there are still some limitations in our study. First, because of the low predictive ability of the features available in SOReg in terms of the Nagelkerke *R*^2^ and AUC [8,9], we failed to diminish overfitting of the DLNN models. We hope to solve this problem by incorporating extra variables on perioperative care in the future. Including these factors is likely to improve the predictive ability; however, these models would not allow guidance in the preoperative setting. Second, although the DLNN models are efficient and able to formulate an adequate solution to the particular question, they are highly specialized to the specific domain, and retraining is usually necessary for the questions that do not pertain to the identical domain [51]. For example, if we want to predict a specific serious complication such as pulmonary embolism after bariatric surgery, we have to modify the layers and readjust hyperparameters in the model because the original models were not trained differentially for the different outcomes. Third, DL requires a large amount of computing power. The high-performance hardwire such as the multicore graphics processing unit is usually needed. It is time consuming and costly, and we have to give up some of the more complicated models because of extreme time inefficiency and leave them for future investigation when more efficient algorithms or more powerful hardware become available.

### Conclusions

Compared with the results from our previous study using traditional ML algorithms to predict the postoperative serious complication after bariatric surgery using SOReg data, the MLP and CNN showed improved, but limited, predictive ability, which deserves further investigation. The overfitting issue is still apparent and needs to be overcome by incorporating more patient features, for example, intra- and perioperative information, from other data resources.

## Appendix

Multimedia Appendix 1 Structure of the MLP model. MLP: multilayer perceptron.

Multimedia Appendix 2 Structure of the CNN model. CNN: convolutional neural network.

Multimedia Appendix 3 Performance of the first 175 MLP models with different computation units, epochs, and batch sizes. In general, performance of the models (measured as AUC) increased with more computation units and epochs, and decreased with larger batch sizes. Although the performance increased with the model’s complexity, the efficiency (measured as AUC divided by logarithmic computing time) decreased. MLP: multilayer perceptron; AUC: area under the curve.

## Abbreviations

1D: one-dimensional
Adam: adaptive moment estimation
AUC: area under curve
CAP: credit assignment path
CNN: convolutional neural network
DLNN: deep learning neural network
HbA1c: hemoglobin A1c
LSTM: long short-term memory
MCC: Matthews correlation coefficient
ML: machine learning
MLP: multilayer perceptron
relu: rectified linear unit
RNN: recurrent neural network
ROC: receiver operating characteristic
SMOTE: synthetic minority oversampling technique
SOReg: the Scandinavian Obesity Surgery Registry
WC: waist circumference
