# Supplementary figures and images for: Deep Learning Neural Networks to Predict Serious Complications After Bariatric Surgery: Analysis of Scandinavian Obesity Surgery Registry Data
Source: JMIR Med Inform. 2020 May 8;8(5):e15992. doi: 10.2196/15992 (PMC7244994; doi:10.2196/15992)

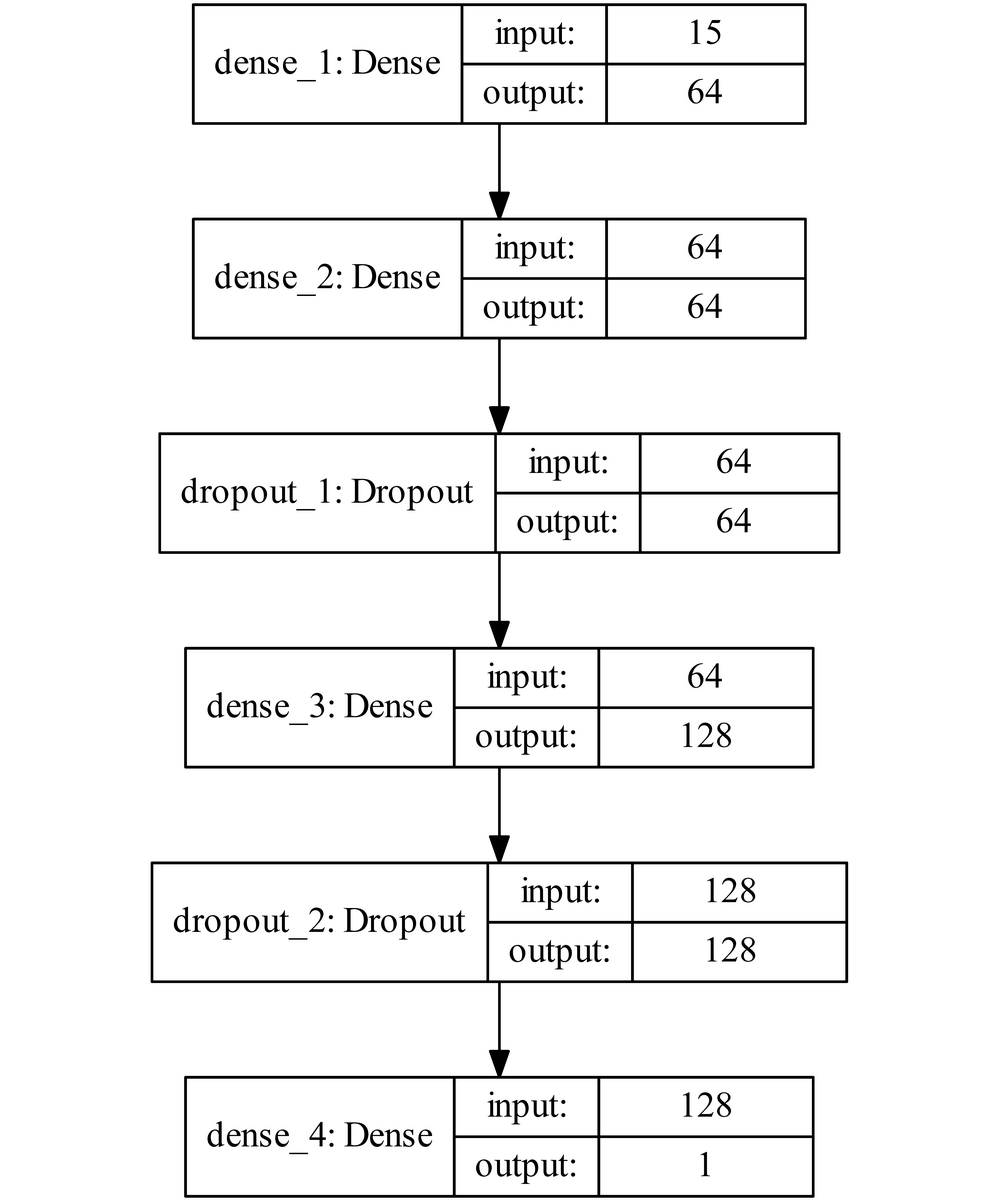

Supplement: Multimedia Appendix 1 [file medinform_v8i5e15992_app1.png]

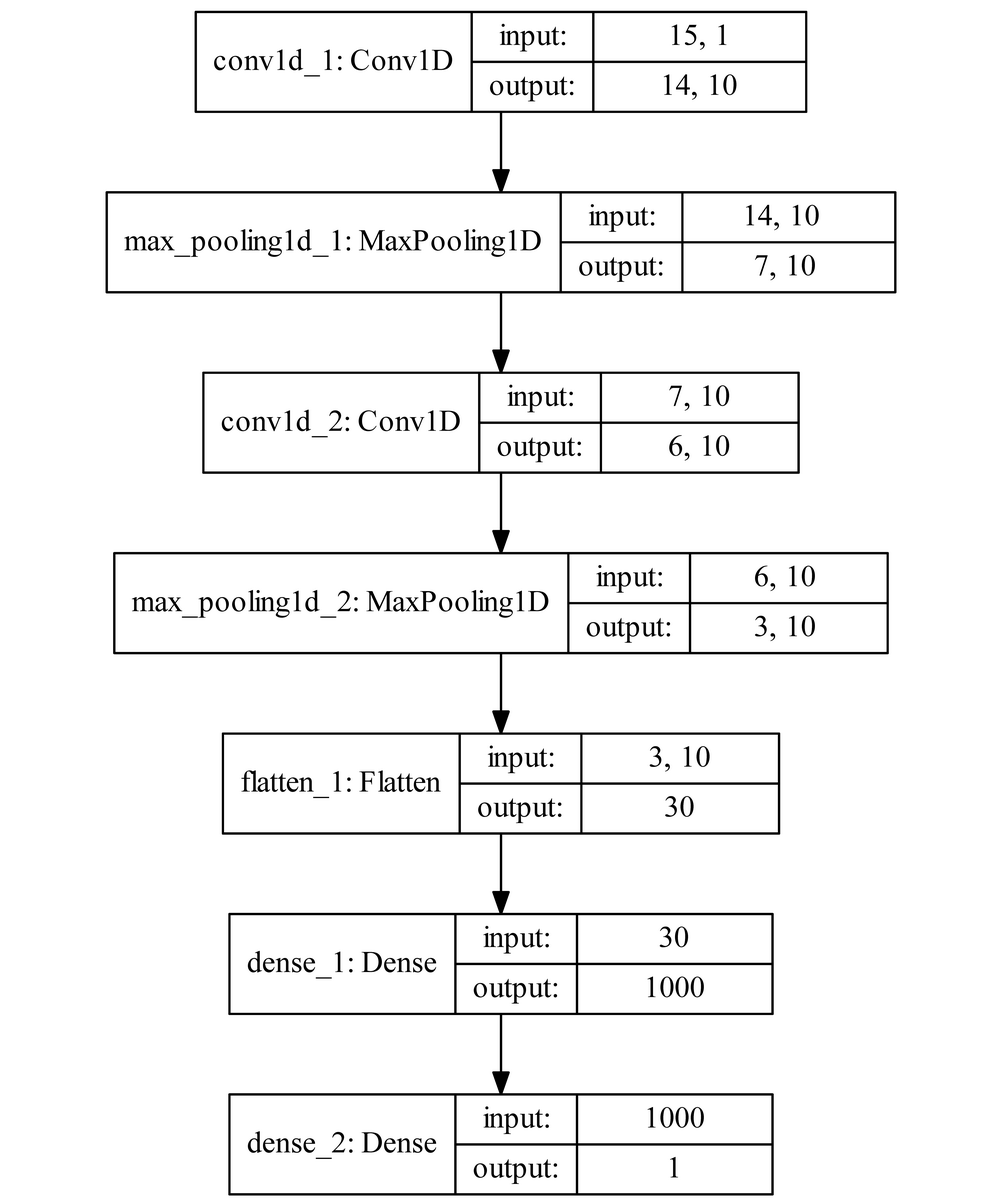

Supplement: Multimedia Appendix 2 [file medinform_v8i5e15992_app2.png]

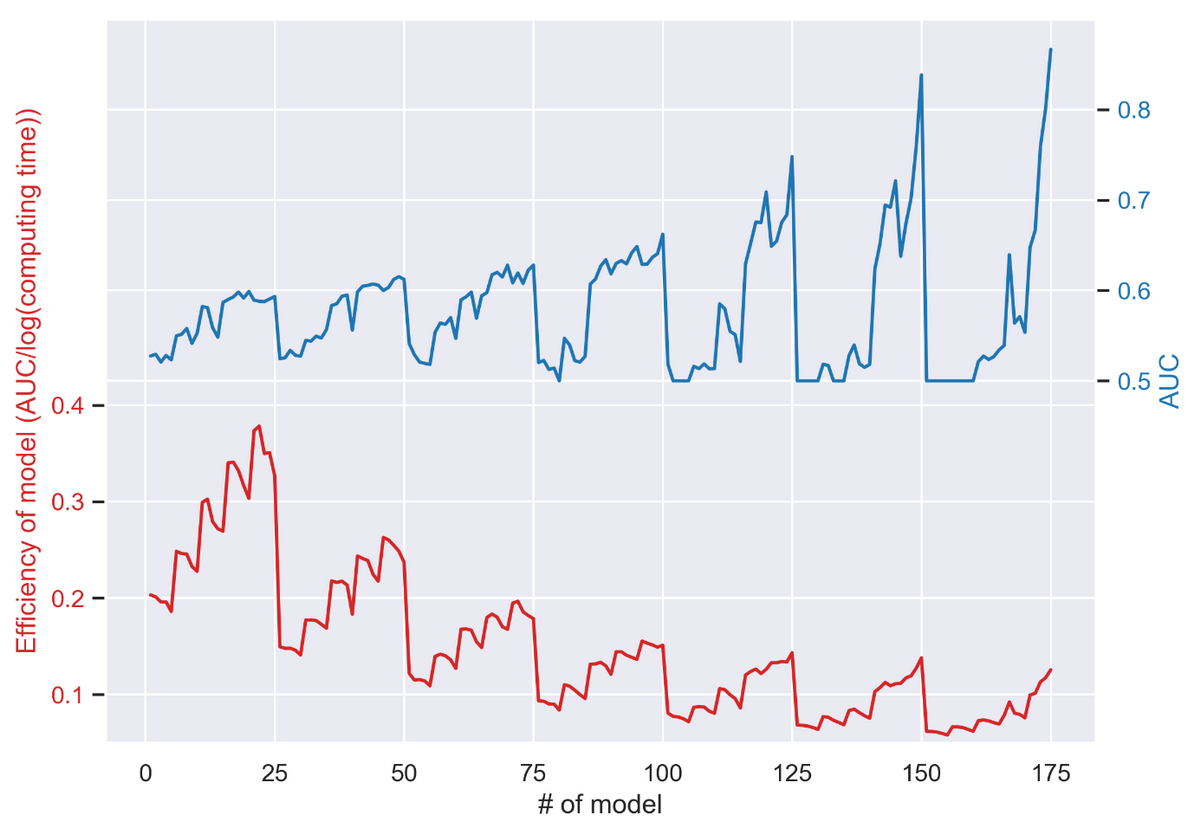

Supplement: Multimedia Appendix 3 [file medinform_v8i5e15992_app3.png]
